# The Association Between Red Cell Distribution Width and Grip Strength in Older Adults

**DOI:** 10.7759/cureus.33049

**Published:** 2022-12-28

**Authors:** Carlos H Orces

**Affiliations:** 1 Rheumatology, Laredo Medical Center, Laredo, USA

**Keywords:** general population, older adults, grip strength, anemia, red cell distribution width

## Abstract

Background

The red cell distribution width (RDW), an index of variation of erythrocyte volume (anisocytosis), has traditionally been used in the differential diagnosis of anemia. However, recent studies reported that increased RDW may be associated with adverse aging-related health outcomes.

Methods

The present cross-sectional study analyzed data from the National Health and Nutrition Examination Survey to examine the association between RDW categories (≤ 13.0%, 13.1 - 14.0%, 14.1 - 15.0%, ≥ 15.1%) and maximum grip strength (GS) (kg) in a nationally representative sample of adults aged 60 years and older. Anemia was defined according to the WHO criteria as a hemoglobin concentration of < 13 g/dl in men and < 12 g/dl in women.

Results

A total of 2,955 participants with a mean age of 69.3 years represented the study sample. General linear models demonstrated that men and women with RDW ≥ 15.1% were 3.2 kg and 1.4 kg weaker than their counterparts with RDW ≤ 13.0%, respectively. Notably, non-anemic older men in the highest RDW category had a mean GS 3.9 kg lower than those in the lowest RDW category. In contrast, this association was attenuated in women without anemia.

Conclusion

RDW was inversely associated with GS, particularly in older men. Moreover, this association remained unchanged even among men without anemia.

## Introduction

Low muscle strength in older adults is associated with adverse health outcomes, including falls, functional decline, and all-cause mortality [1]. Previous population-based studies have consistently demonstrated that low hemoglobin concentrations or prevalent anemia are associated with decreased muscle strength in older adults [2-5]. The red cell distribution width (RDW), an index of variation of erythrocyte volume (anisocytosis), has traditionally been used in the differential diagnosis of anemia. Moreover, increased RDW values may also reflect abnormal erythrocyte homeostasis attributed to underlying cardiometabolic disorders, shortening of telomere length, oxidative stress, inflammation, or poor nutritional status [6].

Notably, Min Kim et al. recently demonstrated among participants in the Osteoporotic fractures in Men (MrOS) Study that elevated RDW values were associated with diverse aging-related outcomes, including decreased grip strength (GS) [7]. The extent to which RDW categories are independently associated with GS among older adults in the general population has not been fully explored. Therefore, the present study aimed to examine the relationship between RDW and GS among older adults in the general population. It was hypothesized that increased RDW would be associated with weaker GS.

## Materials and methods

Study population

The National Health and Nutrition Examination Survey (NHANES) is a continuous biannual study designed to assess the health and nutritional status of adults and children in the United States (U.S.). The NHANES protocol was approved by the National Center for Health Statistics Research Ethics Review Board (study protocol #2011-17). A detailed description of the NHANES methods and analytic guidelines can be found at https://wwwn.cdc.gov/nchs/nhanes/analyticguidelines.aspx.

Covariates

The demographic characteristics of the participants were self-reported. Body mass index (BMI) was calculated as weight in kilograms divided by height in meters squared. Smoking history was categorized as never, former, or current smoker. Participants were also considered to drink alcohol if they responded affirmatively to the question “In any single year, have you had at least 12 drinks of any type of alcoholic beverage?" Self-reported leisure-time physical activity spent in a typical week was calculated and those who met the Physical Activity Guidelines for Americans were considered to be physically active [8].

The estimated Glomerular Filtration Rate (eGFR) was calculated according to the Modification of Diet in Renal Disease formula and subjects with an eGFR < 60 ml/min/1.73 m^2^ were classified as having chronic kidney disease (CKD) [9]. Diabetes mellitus was defined if participants reported a physician diagnosis of diabetes or had a hemoglobin A1c ≥ 6.5%. In addition, a comorbid score was created according to participants’ self-report of a physician's diagnosis of arthritis, congestive heart failure, coronary heart disease, stroke, chronic bronchitis, or cancer.

Serum vitamin B12 (pg/ml) levels were measured with the electrochemiluminescence assay. Moreover, measurements of total testosterone (ng/dl) and 25-hydroxyvitamin D (25(OH)D) levels were performed using isotope dilution and standardized liquid chromatography-tandem mass spectrometry method, respectively. The Beckman Coulter method was used to derive complete blood count (CBC) parameters, in combination with an automatic diluting and mixing device for sample processing, and a single beam photometer for hemoglobinometry. As previously reported, RDW values were classified into four group categories: ≤ 13.0%, 13.1 - 14.0%, 14.1 - 15.0%, and ≥ 15.1% [7]. Anemia was defined according to the WHO criteria as a hemoglobin concentration of < 13 g/dL for men and <12 g/dL for women (https://apps.who.int/iris/bitstream/handle/10665/43894/9789241596657_eng.pdf.).

The muscle strength - grip test component

Muscle strength was measured through a grip test using a Takei digital GS dynamometer, model T.K.K.5401 (Takei Scientific Instruments Co., Ltd., Tokyo, Japan). After the demonstration of the protocol, each hand was tested three times, alternating hands between trials with a 60-second rest between measurements on the same hand. Participants who were able to grip the dynamometer with one hand still performed the test. Detailed descriptions of the GS protocol can be found at https://wwwn.cdc.gov/nchs/data/nhanes/2013-2014/manuals/muscle_strength_2013.pdf.

Older adults with a maximum GS < 35.5 kg for men and < 20 kg for women were considered to have low GS according to the Sarcopenia Definitions and Outcomes Consortium (SDOC) [10].

Statistical analysis

The characteristics of participants were compared across RDW categories using the chi-squared and ANOVA tests for categorical and continuous variables, respectively. Sex-specific general linear models adjusted for demographic and behavioral characteristics, diabetes, number of comorbidities, and biomarkers were assembled to examine the independent association between RDW categories and maximum GS. In a subgroup analysis, maximum GS was examined across RDW categories among older adults according to anemia status. Of 19,931 participants in the NHANES cycles 2011-2012 and 2013-2014, 3,632 subjects were aged 60 years and older. Those with missing data on GS, BMI, and RDW were excluded from this analysis, leaving a study sample of 2,955 participants. Statistical Product and Service Solutions (SPSS) (IBM SPSS Statistics for Windows, Version 25.0, Armonk, NY) was used in all analyses to account for the NHANES complex survey design. Significance was set at a 2-tailed p-value < .05.

## Results

The mean age of participants was 69.3 (SE 0.2) years, and women accounted for 54.3% of the study sample. Table 1 shows the characteristics of participants stratified according to RDW categories. Overall, participants with increased RDW values tended to be older, non-Hispanic black, obese, physically inactive, and had more comorbidities. As expected, a higher prevalence of anemia was seen in both sexes as the RDW categories increased.

**Table 1:** Characteristics of participants according to RDW categories NHW: non-Hispanic white; NHB: non-Hispanic black; eGFR: estimated glomerular filtration rate; BMI: body mass index

	≤ 13.0% (n=966)	13.1 - 14.0% (n=1,223)	14.1 - 15.0% (n=477)	≥ 15.1% (n=289)
Age (years), mean	68.3	69.7	70.1	71.2
Gender, %				
Men	44.6	46.6	51.3	39.7
Women	55.4	53.4	51.3	60.3
Race/ethnicity, %				
Hispanic	7.0	8.2	6.4	5.1
NHW	82.9	79.1	72.8	71.5
NHB	4.2	7.9	15.0	18.8
Others	5.9	4.7	5.8	4.7
BMI (kg/m^2^)	28.1	28.7	30.5	31.1
Smoking status, %				
Never	52.2	50.3	45.6	42.7
Former	37.8	39.4	38.7	44.8
Current	10.0	10.4	15.7	12.5
Alcohol use, %	75.6	70.9	68.0	69.2
Physical activity, %	34.7	28.4	18.4	20.0
Diabetes mellitus, %	14.8	20.0	26.3	27.7
No. comorbidities, %				
0	39.2	30.3	27.8	21.3
1	39.0	42.3	32.0	35.3
2	16.8	21.0	31.1	31.9
≥ 3	5.0	6.3	9.1	11.4
Anemia, %				
Men	5.5	7.4	14.6	48.3
Women	4.3	7.4	19.4	31.2
eGFR(ml/min), mean	74.2	72.8	68.7	66.5
Vitamin B12 (pg/ml)	732.2	706.6	616.8	685.2
Testosterone (ng/dl), mean	192.3	204.8	204.4	155.7
25(OH)D (nmol/L), mean	83.8	82.0	77.5	77.1

As shown in Table 2, after adjusting for potential confounders, older men with RDW values ≥ 15.1% were about 3.2 kg weaker than their counterparts with RDW values ≤ 13.0%. In older women, this association was less accentuated. However, women in the highest RDW category remained 1.4 kg weaker as compared with those in the lowest RDW category.

**Table 2:** Maximum grip strength (kg) according to RDW categories in older adults * P < .05 compared with RDW ≤ 13.0% RDW: red cell distribution width; eGFR: estimated Glomerular Filtration Rate Model 1: adjusted for age, race/ethnicity, and BMI (kg/m^2^) Model 2: adjusted for model 1 and smoking status, alcohol use, physical activity Model 3: adjusted for model 2 and anemia, diabetes, comorbidities, eGFR (ml/min), vitamin B12 (pg/ml), 25(OH)D (nmol/l), and testosterone (ng/dl)

	≤ 13.0% (ref)	13.1 - 14.0%	14.1 - 15.0%	≥ 15.1%	P value
Men					
Model 1	40.7	40.9	39.6	36.5*	< .0001
Model 2	40.7	41.1	40.0	36.7*	< .0001
Model 3	40.6	41.1	40.3	37.4*	< .05
Women					
Model 1	25.3	25.0	24.5	23.3*	< .05
Model 2	25.3	25.1	24.9	23.5*	.071
Model 3	25.2	25.2	25.1	23.8*	.240

As shown in Table 3, the prevalence of low muscle strength in both sexes increased across RDW categories. Notably, older men with RDW values ≥ 15.1% were 2.6 times more likely to have low muscle strength than those with RDW values ≤ 13.0%. Likewise, older women in the highest RDW category had 1.9 higher odds of having low muscle strength than those in the lowest RDW category. However, this association was significantly attenuated after adjusting for behavioral characteristics, number of comorbidities, and biomarkers.

**Table 3:** Associations between RDW categories and low muscle strength in older adults RDW: red cell distribution width; eGFR: estimated Glomerular Filtration Rate a Model 1: adjusted for age, race/ethnicity, and BMI (kg/m^2^) b Model 2: adjusted for model 1 and smoking status, alcohol use, and physical activity c Model 3: adjusted for model 2 and anemia, diabetes, comorbidities, eGFR (ml/min), 25(OH)D (nmol/L), vitamin B12 (pg/ml), and testosterone (ng/dl)

	≤ 13.0% (ref)	13.1 - 14.0%	14.1 - 15.0%	≥ 15.1%
Men				
% (95% CI)	21.2 (16.6 - 26.6)	25.6 (20.2 - 31.9)	34.0 (26.1 - 43.0)	45.1 (33.2 - 57.6)
OR (95% CI)^a^	1.00	1.04 (0.68 - 1.58)	1.60 (0.96 - 2.67)	2.99 (1.57 - 5.70)
OR (95% CI)^b^	1.00	1.03 (0.67 - 1.59)	1.59 (0.91 - 2.76)	3.02 (1.57 - 5.80)
OR (95% CI)^c^	1.00	0.92 (0.57 - 1.46)	1.41 (0.80 - 2.47)	2.67 (1.34 - 5.35)
Women				
% (95% CI)	10.5 (9.1 - 13.4)	14.8 (11.7 - 18.6)	24.4 (19.4 - 30.2)	32.8 (24.8 - 41.9)
OR (95% CI)^a^	1.00	0.81 (0.52 - 1.26)	1.72 (1.11 - 2.65)	1.94 (1.10 - 3.39)
OR (95% CI)^b^	1.00	0.72 (0.45 - 1.15)	1.20 (0.78 – 1.85)	1.60 (0.93 - 2.75)
OR (95% CI)^c^	1.00	0.65 (0.39 - 1.10)	1.06 (0.64 - 1.73)	1.11 (0.63 - 1.96)

**Figure 1:**
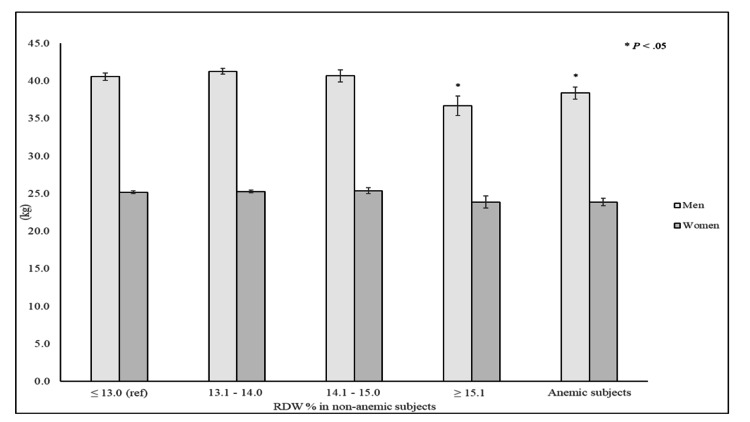
Maximum GS according to RDW categories and anemia status GS: grip strength; RDW: red cell distribution width

Overall, the crude prevalence of anemia was 9.9% in women and 10.6% in men. Of note, non-anemic older men in the highest RDW category and those with prevalent anemia were on average 3.9 kg and 2.2 kg weaker than their counterparts in the lowest RDW category, respectively. In contrast, maximum GS in women did not significantly differ across RDW categories, irrespective of their anemia status (Figure 1).

## Discussion

In a nationally representative sample of older adults, RDW was inversely associated with maximum GS in older adults, particularly in men. Indeed, older men in the highest RDW category were 2.6 times more likely to have low muscle strength than those in the lowest RDW category. In women, this association was significantly attenuated after controlling for potential confounders. Notably, non-anemic men with RDW values ≥ 15.1% and those with anemia were 3.9 kg and 2.2 kg weaker than their non-anemic counterparts with RDW values ≤ 13.0%. The study findings are consistent with those results reported in the MrOS Study in which participants with increased RDW values had significantly weaker GS than those without [7]. Similarly, a recent study conducted among participants in the Korean Urban Rural Elderly study to assess the association between RDW and vertebral fractures demonstrated that older adults in the highest RDW tertile were weaker than their counterparts in the lowest RDW tertile [11].

Although anemia may impair tissue delivery of oxygen, creating local hypoxemia in skeletal muscle and thereby affecting muscle strength, the adverse effect of increased RDW on muscle strength has not been fully elucidated [2,4]. Nevertheless, potential mechanisms might explain this association. Jaiswal et al. initially demonstrated that clonal hematopoiesis, a somatic mutation in hematopoietic stem cells, was associated with several aging-related diseases, particularly cardiometabolic disorders and increased mortality. Of note, the only significant difference found in blood cell indexes was an increased RDW in subjects with single or mutations in more than one gene compared with those without [12]. Likewise, a large proportion of RDW variation has been previously attributed to common genetic variants, which increase with age. Many RDW-related genetic variants have also been reported in aging-related conditions [13]. Lippi et al., in a large cohort of unselected outpatients, described that RDW values were independently and directly associated with inflammatory markers (high-sensitivity C-reactive protein (hs-CRP) and erythrocyte sedimentation rate (ESR)) [14]. Increased inflammatory markers have been associated with decreased skeletal muscle strength in older adults. Thus, it is likely that RDW may be a biomarker, rather than a mediator of low muscle strength [14,15].

The present study has several limitations that should be mentioned. First, the present study results do not necessarily infer causation because of its cross-sectional design. Second, the potential confounder effect of inflammatory biomarkers and iron status on RDW categories was not available for analysis. Despite this limitation, increased RDW values were significantly associated with weaker GS in subjects without anemia. Third, the relationship between RDW and muscle strength was limited to hand GS. It is undetermined whether RDW categories may have similar effects on other muscle groups. Fourth, the study relied on participants' self-reported behavioral characteristics and comorbid conditions, which may have been a source of recall bias.

## Conclusions

RDW was inversely and significantly associated with GS in older men. Notably, this association remained unchanged even in non-anemic older men. Thus, further research is needed to determine whether increased RDW values may represent a biomarker of muscle weakness or have a direct pathophysiologic effect on muscle strength in older adults.
